# Patient-Reported Experiences of Discrimination in the US Health Care System

**DOI:** 10.1001/jamanetworkopen.2020.29650

**Published:** 2020-12-15

**Authors:** Paige Nong, Minakshi Raj, Melissa Creary, Sharon L. R. Kardia, Jodyn E. Platt

**Affiliations:** 1 Department of Health Management and Policy, University of Michigan School of Public Health, Ann Arbor; 2 Department of Kinesiology and Community Health, University of Illinois at Urbana-Champaign, Champaign; 3 Department of Epidemiology, University of Michigan School of Public Health, Ann Arbor; 4 Department of Learning Health Sciences, University of Michigan Medical School, Ann Arbor

## Abstract

**Importance:**

Although considerable evidence exists on the association between negative health outcomes and daily experiences of discrimination, less is known about such experiences in the health care system at the national level. It is critically necessary to measure and address discrimination in the health care system to mitigate harm to patients and as part of the larger ongoing project of responding to health inequities.

**Objectives:**

To (1) identify the national prevalence of patient-reported experiences of discrimination in the health care system, the frequency with which they occur, and the main types of discrimination experienced and (2) examine differences in the prevalence of discrimination across demographic groups.

**Design, Setting, and Participants:**

This cross-sectional national survey fielded online in May 2019 used a general population sample from the National Opinion Research Center’s AmeriSpeak Panel. Surveys were sent to 3253 US adults aged 21 years or older, including oversamples of African American respondents, Hispanic respondents, and respondents with annual household incomes below 200% of the federal poverty level.

**Main Outcomes and Measures:**

Analyses drew on 3 survey items measuring patient-reported experiences of discrimination, the primary types of discrimination experienced, the frequency with which they occurred, and the demographic and health-related characteristics of the respondents. Weighted bivariable and multivariable logistic regressions were conducted to assess associations between experiences of discrimination and several demographic and health-related characteristics.

**Results:**

Of 2137 US adult respondents who completed the survey (66.3% response rate; unweighted 51.0% female; mean [SD] age, 49.6 [16.3] years), 458 (21.4%) reported that they had experienced discrimination in the health care system. After applying weights to generate population-level estimates, most of those who had experienced discrimination (330 [72.0%]) reported experiencing it more than once. Of 458 reporting experiences of discrimination, racial/ethnic discrimination was the most common type (79 [17.3%]), followed by discrimination based on educational or income level (59 [12.9%]), weight (53 [11.6%]), sex (52 [11.4%]), and age (44 [9.6%]). In multivariable analysis, the odds of experiencing discrimination were higher for respondents who identified as female (odds ratio [OR], 1.88; 95% CI, 1.50-2.36) and lower for older respondents (OR, 0.98; 95% CI, 0.98-0.99), respondents earning at least $50 000 in annual household income (OR, 0.76; 95% CI, 0.60-0.95), and those reporting good (OR, 0.59; 95% CI, 0.46-0.75) or excellent (OR, 0.41; 95% CI, 0.31-0.56) health compared with poor or fair health.

**Conclusions and Relevance:**

The results of this study suggest that experiences of discrimination in the health care system appear more common than previously recognized and deserve considerable attention. These findings contribute to understanding of the scale at which interpersonal discrimination occurs in the US health care system and provide crucial evidence for next steps in assessing the risks and consequences of such discrimination. The findings also point to a need for further analysis of how interpersonal discrimination interacts with structural inequities and social determinants of health to build effective responses.

## Key Points

**Question:**

What are the national prevalence, frequency, and main types of discrimination that adult patients report experiencing in the US health care system?

**Findings:**

In this nationally representative cross-sectional survey study, 21% of 2137 US adult survey respondents indicated that they had experienced discrimination in the health care system, and 72% of those who had experienced discrimination reported experiencing it more than once. Racial/ethnic discrimination was the most frequently reported type of discrimination respondents experienced.

**Meaning:**

Experiences of discrimination in the health care system appear to be more common than previously recognized and deserve considerable attention.

## Teaser

This cross-sectional study examines the responses to a recent National Opinion Research Center survey to assess the prevalence, frequency, and main types of discrimination experienced by adult patients in the US health care system.

## Introduction

Health systems in the US are increasingly expressing concern about understanding and responding to social determinants of health (ie, the social and environmental conditions that may influence individual health and the differences in health and health outcomes between groups).^1,2,3^ Considerable analytical work has identified a range of factors associated with inequities in treatments, outcomes, and mortality across race, sex, socioeconomic status, and various other social identities.^1,4,5,6,7,8,9,10,11^ Some of these include patient-clinician discordance, physician bias, and daily experiences of discrimination.^1,3,12,13^ Daily experiences of discrimination in other contexts (eg, while shopping, in employment, or in housing) have been studied extensively in association with downstream health outcomes, including but not limited to hypertension, cardiovascular disease, poor sleep, mental health symptoms, lower trust in the health care system, delayed or avoided care, and underuse of mental health services.^14,15,16,17,18,19,20^ Despite considerable knowledge about the association between discrimination and health care utilization rates and health outcomes and the relevance of discrimination to health inequity, to our knowledge, experiences of discrimination in the health care system itself are understudied.

More specifically, previous work has provided important insights regarding the association between discrimination and health but has not identified patient-reported lifetime experiences of discrimination in the health care system at a national level in a way that captures the frequency and that allows for a full self-selection of the types of discrimination experienced. For example, some studies have drawn from narrow regional samples or limited respondent reports to the previous 12 months,^19,21,22,23^ whereas other studies have asked participants to report discrimination associated with a single aspect of their identity, such as race or sex, as preselected by the research team.^24,25^ In addition, there is limited information on the frequency of different types of discriminatory treatment, which may be a significant risk factor for chronic disease given the association between discrimination and health over the life course.^26,27^

To better understand and respond to interpersonal discrimination in the health care system, as well as the potential downstream effects of discrimination in the context of structural inequity, it is necessary to identify these experiences and the frequency with which they occur at the national level. The objective of our study was to characterize patient-reported experiences of discrimination in a nationally representative sample of the US population in terms of (1) prevalence, (2) primary types of discrimination, and (3) frequency. To our knowledge, this is the first study to examine the prevalence, frequency, and types of discrimination in the health care system using a nationally representative sample that does not limit the respondents’ reporting time frame to 1 year or less.

## Methods

### Sample

We used the National Opinion Research Center (NORC) AmeriSpeak Panel probability-based, nationally representative sample of English-speaking US adults to conduct an online survey in May 2019. Prior to data collection, the survey instrument was pretested (n = 320). The research team conducted 17 cognitive interviews to assess comprehension and to improve the clarity of the survey questions, and NORC conducted a pilot survey with 115 respondents. Of 3253 surveys sent, 2157 individuals responded and completed the final survey (for a response rate of 66.3%) after being recruited via the NORC panel. We oversampled African American respondents, Hispanic respondents, and respondents with annual household incomes below 200% of the federal poverty level. Poststratification survey weights were calculated by NORC based on age, sex, educational level, race/ethnicity, housing tenure, telephone status, and Census division from the Current Population Survey. They also included weights for survey nonresponse (eAppendix in the Supplement). This study followed the American Association for Public Opinion Research (AAPOR) reporting guideline for survey studies. The institutional review board of the University of Michigan reviewed and approved this project and waived the requirement to obtain informed consent because the research involved no more than minimal risk to participants, who had already provided informed consent to NORC.

### Measures

To assess experiences of discrimination, we adapted the Major Experiences of Discrimination measures and the Experiences of Discrimination measures from the Coronary Artery Risk Development in Young Adults study.^28,29^ We asked respondents (1) whether they had ever been discriminated against, hassled, or made to feel inferior while getting medical care and, if so, (2) what they believed was the main reason for that experience, and (3) how frequently they experienced this discrimination. A response of “yes” to the first question was defined as an experience of discrimination. Respondents chose from a list of 13 potential reasons for the discrimination, adapted from the Major Experiences of Discrimination measures, including an open-ended response for other reasons not listed. Two research team members (P.N. and M.C.) separately coded the free-text responses under “other” and reconciled any differences with a third team member (M.R.). Those responses were classified under extant categories or under additional categories that emerged through thematic analysis. Remaining free-text responses retained the “other” designation if they remained miscellaneous. After coding, there were 18 total types of discrimination for analysis.

The survey instrument defined the health care system as “the healthcare professionals and institutions that you personally interact with when getting health care.” Respondents self-reported their sex and racial or ethnic identity and reported their current insurance status as a binary measure of whether they currently had health insurance. They also indicated when they last received medical care and reported their health status on a 5-point scale ranging from poor to excellent health. We excluded 20 observations that had missing data for any of the measures included in the analysis.

### Statistical Analysis

We analyzed survey responses from 2137 participants with complete data. We first compared respondents who had experienced discrimination in the health care system with those who had not, using various demographic measures, including sex, age, race/ethnicity, educational level, income, health insurance status, rural or urban residency, having a regular source of medical care, having received care in the last 12 months, and self-reported health status. We conducted weighted bivariable and multivariable logistic regressions to examine associations between these variables and reported experiences of discrimination. We defined statistical significance as *P* < .05 in 2-tailed tests. Next, we enumerated the reported types of discrimination, identified the most commonly reported types of discrimination, and then assessed the frequency of experiencing the 5 most commonly given reasons for discrimination. All reported percentages are weighted to provide population estimates. All analyses were conducted with Stata, version 14 (StataCorp).

## Results

Table 1 summarizes the demographic characteristics and general health status of all 2137 survey respondents and of the 458 respondents who reported experiences of discrimination, with unweighted frequencies and weighted percentages. Based on weighted percentages, approximately half of all respondents (1047 [52.3%]) were male (unweighted, 51.0% female). The mean (SD) age of respondents was 49.6 (16.3) years (range, 21.0-91.0 years), and the sample reflected the racial/ethnic composition of the US.^30^ The majority of respondents had at least some college education (1675 [77.9%]), and approximately half (1022 [50.2%]) earned at least $50 000 in annual household income. Most respondents had health insurance (1890 [89.4%]) and lived in metropolitan areas (1899 [89.3%]). A large majority of respondents reported receiving care in the last 12 months (1809 [85.3%]) and having a regular source of care (1708 [81.0%]). Overall, 916 respondents (43.3%) reported being in good health. Just over one-fifth of respondents (458 [21.4%]; SE, 0.009) reported that they had experienced discrimination while getting medical care. The majority of respondents reporting discrimination were female (289 [63.1%]) and reported less than $50 000 in annual household income (279 [60.9%]). Compared with non-Hispanic White respondents (252 [20.3%]), higher proportions of Hispanic respondents (96 [22.9%]), non-Hispanic Black respondents (77 [22.8%]), and non-Hispanic respondents with other racial/ethnic identities (33 [23.4%]) reported experiences of discrimination (eTable in the Supplement).

**Table 1.** Characteristics of 2137 Respondents, a US Nationally Representative Sample, Who Reported Experiencing Discrimination

Characteristic	Respondents, No. (weighted %)	
	Full sample	Respondents who experienced discrimination
Total	2137 (100)	458 (21.4)
Sex		
Female	1090 (47.7)	289 (63.1)
Male	1047 (52.3)	169 (36.9)
Age, mean (SD), y	49.6 (16.3)	46.0 (15.0)
Educational level		
No high school diploma	81 (3.7)	19 (4.1)
High school equivalent	381 (18.4)	72 (15.7)
Some college	985 (44.7)	234 (51.1)
Bachelor’s degree or above	690 (33.2)	133 (29.0)
Race/ethnicity		
Non-Hispanic White	1239 (58.8)	252 (55.0)
Non-Hispanic Black	338 (15.5)	77 (16.8)
Hispanic	419 (19.2)	96 (21.0)
Non-Hispanic other^a^	141 (6.4)	33 (17.2)
Household income, $		
<50 000	1115 (49.8)	279 (60.9)
≥50 000	1022 (50.2)	179 (39.1)
Insurance coverage		
No	247 (10.6)	69 (15.1)
Yes	1890 (89.4)	389 (84.9)
Metropolitan residence		
Yes	1899 (89.3)	399 (87.1)
No	238 (10.7)	59 (12.9)
Has a regular source of care		
No	429 (19.0)	110 (24.0)
Yes	1708 (81.0)	348 (76.0)
Received care in the last 12 mo		
No	328 (14.7)	81 (17.7)
Yes	1809 (85.3)	377 (82.3)
Self-reported health		
Poor or fair	550 (22.7)	169 (36.9)
Good	916 (43.3)	189 (41.3)
Very good or excellent	671 (34.0)	100 (21.8)

^a^ Includes self-selected non-Hispanic other (n = 36), non-Hispanic multiracial (n = 51), and non-Hispanic Asian (n = 54).

We observed statistically significant differences in reported experiences of discrimination across demographic groups and health-related characteristics (Table 2). In bivariable analysis, those more likely to experience discrimination were female (odds ratio [OR], 1.87; 95% CI, 1.52-2.32), had poor or fair self-reported health status (OR, 1.71; 95% CI, 1.34-2.17 compared with good health), or lacked health insurance (OR, 1.50; 95% CI, 1.11-2.02). Those less likely to experience discrimination had an annual household income of at least $50 000 (OR, 0.64; 95% CI, 0.52-0.79), were older (OR, 0.98; 95% CI, 0.98-0.99), or had a regular source of medical care (OR, 0.74; 95% CI, 0.58-0.95). In multivariable analysis, these associations remained statistically significant with the exception of having a regular source of care (OR, 0.91; 95% CI, 0.68-1.14) and insurance coverage (OR, 1.21; 95% CI, 0.87-1.68). In multivariable analysis, the odds of experiencing discrimination were higher for respondents who identified as female (OR, 1.88; 95% CI, 1.50-2.36) and lower for older respondents (OR, 0.98; 95% CI, 0.98-0.99), respondents earning at least $50 000 in annual household income (OR, 0.76; 95% CI, 0.60-0.95), and those reporting good (OR, 0.59; 95% CI, 0.46-0.75) or excellent (OR, 0.41; 95% CI, 0.31-0.56) health compared with poor or fair health.

**Table 2.** Weighted Bivariable and Multivariable Logistic Regression of Experiences of Discrimination Associated With Demographic and Health Characteristics of 2137 US Residents

Characteristic	Bivariable regression		Multivariable regression	
	OR (95% CI)	*P* value	OR (95% CI)	*P* value
Sex				
Male	1 [Reference]		1 [Reference]	
Female	1.87 (1.52-2.32)	<.001	1.88 (1.50-2.36)	<.001
Age, mean (SD), y	0.98 (0.98-0.99)	<.001	0.98 (0.98-0.99)	<.001
Educational level				
No high school diploma	1 [Reference]		1 [Reference]	
High school equivalent	0.76 (0.43-1.4)	.35	0.87 (0.47-1.6)	.66
Some college	1.02 (0.60-1.74)	.95	1.30 (0.73-2.31)	.37
Bachelor’s degree or above	0.78 (0.45-1.35)	.37	1.23 (0.68-2.25)	.49
Race/ethnicity				
Non-Hispanic White	1 [Reference]		1 [Reference]	
Non-Hispanic Black	1.16 (0.87-1.54)	.33	0.93 (0.68-1.28)	.57
Hispanic	1.16 (0.89-1.52)	.26	0.86 (0.64-1.16)	.31
Non-Hispanic other^a^	1.20 (0.79-1.81)	.39	0.973 (0.64-1.49)	.90
Household income, $				
<50 000	1 [Reference]		1 [Reference]	
≥50 000	0.64 (0.52-0.79)	<.001	0.76 (0.60-0.95)	.02
Insurance coverage				
Yes	1 [Reference]		1 [Reference]	
No	1.50 (1.11-2.02)	.008	1.21 (0.87-1.68)	.25
Metropolitan residence				
No	1 [Reference]		1 [Reference]	
Yes	0.81 (0.59-1.11)	.18	0.82 (0.59-1.20)	.24
Has a regular source of care				
No	1 [Reference]		1 [Reference]	
Yes	0.74 (0.58-0.95)	.02	0.91 (0.68-1.14)	.47
Received care in the last 12 mo				
No	1 [Reference]		1 [Reference]	
Yes	0.80 (0.61-1.06)	.118	0.91 (0.66-1.26)	.560
Self-reported health				
Poor or fair	1 [Reference]		1 [Reference]	
Good	0.59 (0.46-0.75)	<.001	0.59 (0.46-0.75)	<.001
Very good or excellent	0.395 (0.30-0.52)	<.001	0.41 (0.31-0.56)	<.001

Abbreviation: OR, odds ratio.

^a^ Includes self-selected non-Hispanic other (n = 36), non-Hispanic multiracial (n = 51), and non-Hispanic Asian (n = 54).

The 5 most commonly reported types of discrimination among 458 respondents were based on race/ethnicity (79 [17.2%]), educational or income level (59 [12.9%]), weight (53 [11.6%]), sex (52 [11.4%]), and age (44 [9.6%]). Just over one-quarter of respondents reporting discrimination selected “other reasons” for discrimination (121 [26.4%]). After coding free-text responses, some of which overlapped with extant categories, we identified 6 additional types of discrimination. The most common of these included insurance and health finances or ability to pay for care (21 [4.6%]). One respondent described this type of discrimination by writing “I felt that with Medicaid [you] get pushed aside but when I had Blue Cross Blue Shield I [was seen] immediately.” Drug use and medication use were also sources of discrimination for some respondents (18 [3.9%]). This category referred to stigma and discrimination based on the medications that respondents were taking, prior substance use, or assumed drug-seeking behavior. For example, 1 respondent reported that “I was honest about having a drug addiction. They treated me like I was not important at all and insinuated that I was just trying to get pills.”

Discrimination based on mental health status was also reported by 9 respondents (2.0%) in free-text responses, and lifestyle (eg, having tattoos) was reported by 5 respondents (1.1%). Forty-two respondents (9.2%) who reported discrimination felt hassled or discriminated against because of their clinician’s attitude or behavior. This included feeling dismissed or disrespected by clinicians in a way that was not captured by the multiple-choice responses. Responses coded as “provider attitude” reflected comments that described experiences of being treated poorly, disbelieved, or brushed off while seeking care. Reasons that remained miscellaneous retained the “other” label (18 [3.9%]). Table 3 gives the frequencies for the primary types of discrimination that respondents reported and includes all reported reasons for discrimination as selected by respondents for the entire sample and by race. Although racial/ethnic discrimination was the most commonly reported type of discrimination, race/ethnicity was not significant in the bivariable or multivariable analysis. This is a statistical power issue because non-Hispanic White respondents, predominant in the sample, reported far less racial discrimination (10 [4.0%]) than non-Hispanic Black (42 [54.6%]), Hispanic (21 [21.9%]), and other racial and ethnic minority (6 [18.2%]) respondents. Table 3 also gives the differences in proportions of respondents reporting discrimination by race.

**Table 3.** Frequencies and Weighted Percentages of Reasons for Experiences of Discrimination by Race/Ethnicity

Reason for experience of discrimination	Respondents, No. (weighted %)				
	Total (N = 458)	Non-Hispanic other (n = 33)	Hispanic (n = 96)	Non-Hispanic Black (n = 77)	Non-Hispanic White (n = 252)
Race/ethnicity	79 (17.3)	6 (18.2)	21 (21.9)	42 (54.6)	10 (4.0)
Educational or income level	59 (12.9)	6 (18.2)	13 (13.5)	5 (6.5)	35 (13.9)
Weight	53 (11.6)	5 (15.2)	9 (9.4)	4 (5.2)	35 (13.9)
Sex	52 (11.4)	3 (9.1)	9 (9.4)	6 (7.8)	34 (13.5)
Age	44 (9.6)	1 (3.0)	8 (8.3)	3 (3.9)	32 (12.7)
Clinician attitude or behavior	42 (9.2)	2 (6.1)	1 (1.0)	2 (2.6)	37 (14.7)
Insurance and health finances	21 (4.6)	1 (3.0)	5 (5.2)	4 (5.2)	11 (4.4)
Drug or medication use	18 (3.9)	1 (3.0)	3 (3.1)	0 (0)	14 (5.5)
Physical disability	18 (3.9)	2 (6.1)	4 (4.2)	2 (2.6)	10 (4.0)
Sexual orientation	12 (2.6)	0	4 (4.2)	1 (1.3)	7 (2.8)
Shade of skin color	10 (2.2)	3 (9.1)	4 (4.2)	3 (3.9)	0 (0)
Mental health status	9 (2.0)	1 (3.0)	1 (1.0)	0 (0)	7 (2.8)
Ancestry or national origin	6 (1.3)	1 (3.0)	4 (4.2)	1 (1.3)	0 (0)
Speaking English as a second language	6 (1.3)	1 (3.3)	5 (5.2)	0 (0)	0 (0)
Religion	5 (1.1)	0 (0)	3 (3.1)	0 (0)	2 (0.8)
Lifestyle	5 (1.1)	0 (0)	0 (0)	0 (0)	5 (2.0)
Height	1 (0.2)	0 (0)	0 (0)	0 (0)	1 (0.4)
Other	18 (3.9)	0 (0)	2 (2.1)	4 (5.2)	12 (4.8)

Among 458 respondents who reported discrimination in the health care system, 330 (72.1%) said that they had experienced it more than once. We report the frequency of these experiences in Table 4. The majority of respondents who experienced discrimination across all 5 of the most commonly reported types of discrimination reported experiencing it 2 or 3 times. In fact, 16 respondents (20.3%) who experienced racial discrimination and 13 respondents (22.0%) who experienced discrimination based on their educational or income level experienced it 4 or more times. Sex (5 respondents [9.6%]) and age discrimination (3 respondents [6.8%]) were less frequently reported as occurring 4 or more times.

**Table 4.** Weighted Frequency of Experiencing Discrimination Among the 5 Most Common Types of Discrimination

Frequency, No. of experiences	Respondents, No. (weighted %)					
	Total (N = 458)	Reasons reported by at least 25 respondents				
		Race	Educational or income level	Weight	Sex	Age
1	128 (28.0)	20 (25.3)	14 (23.7)	13 (24.5)	16 (30.8)	15 (34.1)
2 or 3	247 (54.0)	43 (54.4)	32 (54.2)	32 (60.4)	31 (59.6)	26 (59.1)
≥4	83 (18.1)	16 (20.3)	13 (22.0)	8 (15.1)	5 (9.6)	3 (6.8)

## Discussion

Our study estimates that, overall, more than 1 in 5 adults in the US have experienced discrimination at least once while receiving health care. Racial discrimination was the most commonly reported type of discrimination, followed by discrimination based on educational or income level, weight, sex, and age. After conducting multivariable logistic regressions, we found that respondents who were younger, identified as female, had lower annual household income, and reported poor or fair health were statistically significantly more likely to report experiences of discrimination.

Our results are consistent with previous studies examining experiences of discrimination in health care as well as in other settings. For example, prior work has found between 25.2% and 43.5% of survey respondents reporting ever experiencing discrimination in any setting.^31^ Estimates of discrimination in the health care system have varied based on the use of different reporting time frames and sampling approaches. For example, one national study of discrimination found that 7.3% of respondents had experienced discrimination in the health care system only in the previous 12 months, whereas a community survey found approximately 14% of respondents reported ever experiencing discrimination in the health care system. This proportion was higher for Black respondents and Latino respondents, which was also true in our sample.^24,28^ Although there may be a lower prevalence of discrimination in the health care system compared with some other settings, such as housing or policing, discrimination is still a frequent experience among patients, and health care is not immune to larger national trends.^28,29^ Health care settings are also distinct; for instance, patients may be more forgiving or may not recall discriminatory incidents after a visit if they were very concerned about a serious illness. Patient experiences of discrimination may actually be higher and require further study through mixed-methods approaches.

Experiences of discrimination in the health care system harm patients by negatively impacting trust, communication, and health-seeking behaviors.^13,16,32,33^ Our findings underscore the importance of understanding aspects of patient identity, especially with regard to race/ethnicity, not as risk factors for discrimination or the downstream effects of those experiences; rather, exposure to discrimination and racism are the risks.^34,35^ The prevalence of discrimination identified in this study points to a need to examine discrimination in the health care system as a risk factor for other negative effects. Future work on interpersonal discrimination in the health care system should examine the types of discrimination we have identified herein, with the understanding that they are harms imposed on patients rather than caused by or reflective of patient demographic characteristics.^36^ This future work should also explore the ways that discrimination is manifested and where in the health care system it is occurring most often.

Our study analyzes a wide variety of types of discrimination, including several highlighted directly by respondents. Each of these types of discrimination requires focused analysis and particular policy responses. For example, in supplementary analyses (eFigure 2 in the Supplement), most of the respondents experiencing racial discrimination were Black persons. To effectively respond to the harms of racial discrimination in the health care system, anti-Black racism needs to be specifically analyzed and addressed. Furthermore, recognizing that discrimination is not discrete or necessarily additive, future work should also inform policy responses by building on existing literature to investigate the effects of layered or interacting types of interpersonal discrimination. For example, the vast majority of respondents reporting weight-based discrimination in the present study were women (eFigure 1 in the Supplement). The intersection between sex- and weight-based discrimination represents only 1 example of how policy will need to respond to intersections of identity and discrimination. Intersectional policy and practice guidance will need to be built on and responsive to these multiple dimensions of discrimination to effectively respond to them.^37^ Such work should complement and inform efforts to address systemic inequities.

Patient self-reports of discrimination are challenging to measure because the specific types of discrimination occurring may be unclear. This survey was able to capture only a single type of discrimination, which may mean that the reports underestimated patient experiences of discrimination. Some of these discriminatory experiences may also be internalized and denied, which suggests that reports may further underestimate the prevalence of discrimination.^38^ Nevertheless, patient perspectives are critical in analyses and in policy designs that aim to address discrimination and health inequities. The prevalence of discrimination in the health care system that we identified builds on existing evidence that it is a problem requiring large-scale policy responses. As health care institutions throughout the country reckon with how systems interact to produce inequity, our study provides national estimates of the prevalence, frequency, and types of discrimination useful for policy, serving as 1 step in the process of building evidence-based responses.

In addition to a broad reckoning with discrimination, there are also localized approaches that may be appropriate to reduce harm in the shorter term. These include seeking information about experiences of discrimination and using that information to alter system-level policies to address inequality.^39^ Health care systems can include measures of experiences of discrimination in their patient surveys to identify the occurrence of discrimination in their organizations and its effects on their patient populations to respond appropriately. Our study may provide guidance on the types of questions to include because the survey items used here build on previously validated measures. Furthermore, our analysis of “other” types of discrimination may suggest additional categories for inclusion in patient surveys. For example, the frequency with which medication or drug use and insurance-based discrimination were reported in this study indicates that further analysis of these health-specific types of discrimination may be warranted. This expanded data collection will enable health care systems to identify the particular types of discrimination occurring in their organizations and, most importantly, address them systematically.

### Limitations

There are a few limitations to this study that should be considered in interpreting the results. The survey questions allowed participants to report only 1 primary type of discrimination, which limits our understanding of the multidimensionality of discrimination and the nature of encounters during which experiences of discrimination occurred.^40,41^ Furthermore, self-reports of discrimination are challenging to measure because some of these experiences may be internalized and denied by individuals, making our estimate of approximately 20% of people who have ever experienced discrimination while receiving health care potentially underestimated.^38^ Also contributing to a potential underestimation are the limits of race/ethnicity response categories that did not specifically capture American Indian, Alaska Native, or Middle Eastern identities. The survey was conducted only in English, which may have excluded some potential respondents. Finally, although we include some supplementary analyses that began to analyze across multiple demographic categories and types of discrimination, future analysis will need to address these numerous dimensions of identity and discrimination in more detail.

## Conclusions

This is the first study, to our knowledge, that has examined the prevalence, frequency, and types of discrimination in the health care system using a nationally representative sample without limiting respondents’ reporting time frame. We found that experiences of discrimination in the health care system (21.4%) were more common than previously known and that these experiences typically occurred more than once. The 5 most commonly reported primary types of discrimination that we identified were based on race/ethnicity, educational or income level, weight, sex, and age. Addressing the immediate harms of these types of discrimination in the health care system should be an immediate policy and health care system priority.

## Notes

Supplement. **eTable.** Weighted Row Percentages of Descriptive Statistics (n = 2,137) **eFigure 1.** Five Most Common Reasons for Experiencing Discrimination, by Gender **eFigure 2.** Five Most Common Reasons for Experiencing Discrimination, by Race **eAppendix.** Sampling and Recruitment
