# Supplementary material for: Patient-Reported Experiences of Discrimination in the US Health Care System
Source: JAMA Netw Open. 2020 Dec 15;3(12):e2029650. doi: 10.1001/jamanetworkopen.2020.29650 (PMC7739133; doi:10.1001/jamanetworkopen.2020.29650)
Supplement: Supplement. — eTable. Weighted Row Percentages of Descriptive Statistics (n = 2,137) eFigure 1. Five Most Common Reasons for Experiencing Discrimination, by Gender eFigure 2. Five Most Common Reasons for Experiencing Discrimination, by Race eAppendix. Sampling and Recruitment [file jamanetwopen-e2029650-s001.pdf]

Supplementary Online Content

Nong P, Raj M, Creary M, Kardia SLR, Platt JE. Patient-reported experiences of discrimination in the US health care system. *JAMA Netw Open*. 2020;3(12):e2029650. doi:10.1001/jamanetworkopen.2020.29650

eTable. Weighted Row Percentages of Descriptive Statistics (n=2,137)
eFigure 1. Five Most Common Reasons for Experiencing Discrimination, by Gender
eFigure 2. Five Most Common Reasons for Experiencing Discrimination, by Race
eAppendix. Sampling and Recruitment

This supplementary material has been provided by the authors to give readers additional information about their work.

© 2020 Nong P et al. *JAMA Network Open*.

eTable. Weighted Row Percentages of Descriptive Statistics (n=2,137)

	Respondents who have experienced discrimination n (weighted %)	Respondents who have not experienced discrimination n (weighted %)
Total	458 (21.43)	1679 (78.57)
Sex		
Female	289 (26.51)	801 (73.49)
Male	169 (16.14)	878 (83.86)
Age (years)		
mean (sd)	45.95 (15.04)	50.60 (16.47)
Education		
No high school diploma	19 (23.46)	62 (76.54)
High school equivalent	72 (18.90)	309 (81.10)
Some college	234 (23.76)	751 (76.24)
BA or above	133 (19.28)	557 (80.72)
Race/Ethnicity		
White, non-Hispanic	252 (20.34)	987 (79.66)
Black, non-Hispanic	77 (22.78)	261 (77.22)
Hispanic	96 (22.91)	323 (77.09)
Other, non-Hispanic*	33 (23.40)	108 (76.60)
Household Income		
< $50,000	279 (25.02)	836 (74.98)
$50,000 +	179 (17.51)	843 (82.49)
Insurance Coverage		
No	69 (27.94)	178 (72.06)
Yes	389 (20.58)	1501 (79.42)
Metropolitan Area		
Metro	399 (21.01)	1500 (78.99)
Non-Metro	59 (24.79)	179 (75.21)
Has a regular source of care		
No	110 (25.64)	319 (74.36)
Yes	348 (20.37)	1360 (79.63)
Received care in the last 12 months		
No	81 (24.70)	247 (75.30)
Yes	377 (20.84)	1432 (79.16)

© 2020 Nong P et al. *JAMA Network Open*.

Self-reported health		
Poor/fair	169 (30.73)	381 (69.27)
Good	189 (20.63)	727 (79.37)
Very good/excellent	100 (14.90)	571 (85.10)

© 2020 Nong P et al. *JAMA Network Open*.

eFigure 1. Five Most Common Reasons for Experiencing Discrimination, by Gender

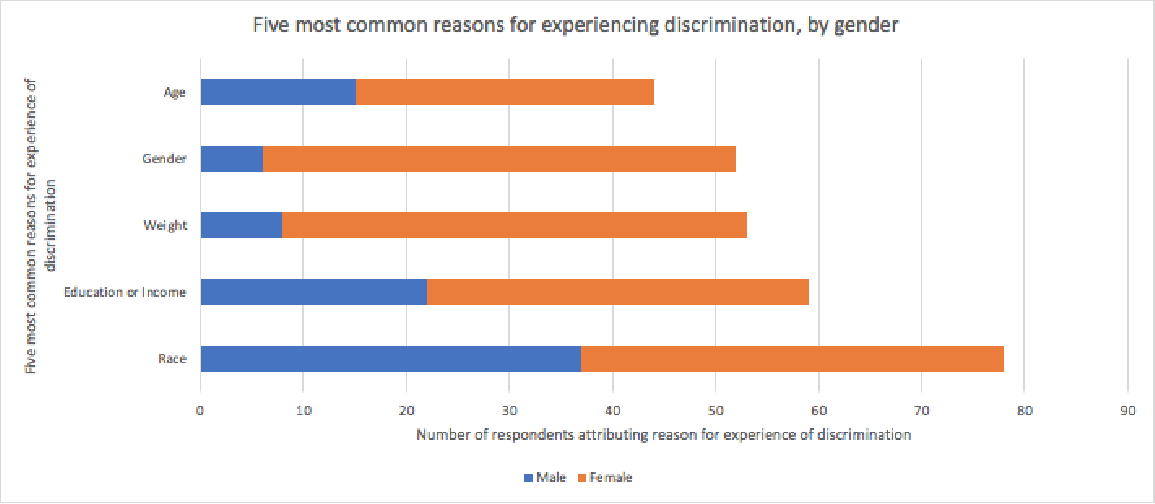

© 2020 Nong P et al. *JAMA Network Open*.

eFigure 2. Five Most Common Reasons for Experiencing Discrimination, by Race

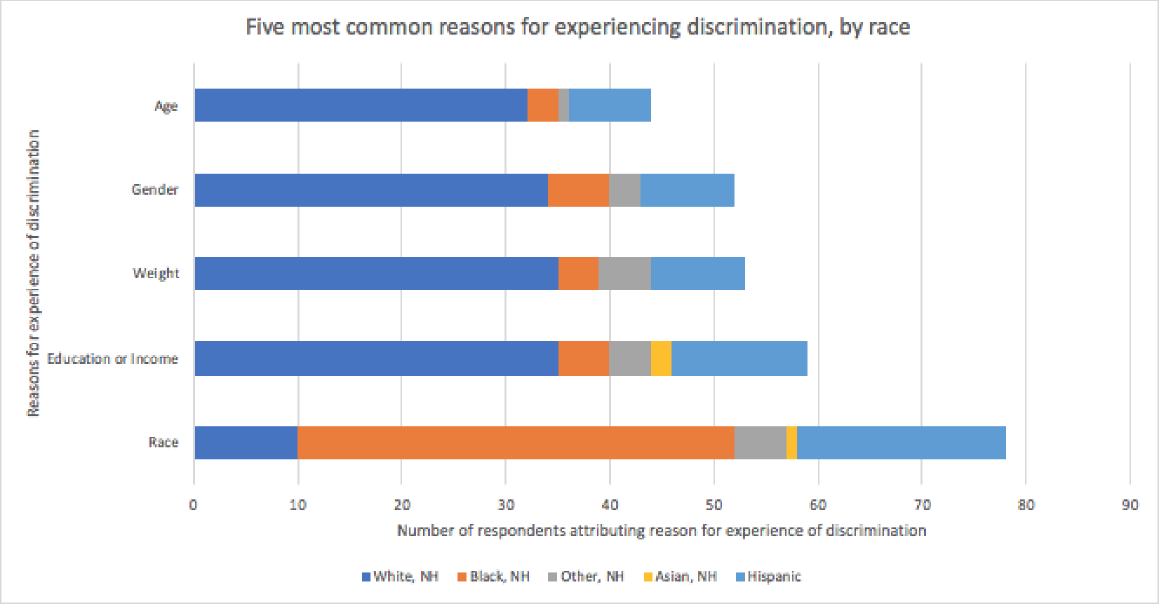

© 2020 Nong P et al. *JAMA Network Open*.

eAppendix. Sampling and Recruitment

Funded and operated by NORC at the University of Chicago, AmeriSpeak® is a probability-based panel designed to be representative of the US household population. Randomly selected US households are sampled using area probability and address-based sampling, with a known, non-zero probability of selection from the NORC National Sample Frame. These sampled households are then contacted by US mail, telephone, and field interviewers (face to face). The panel provides sample coverage of approximately 97% of the U.S. household population. Those excluded from the sample include people with P.O. Box only addresses, some addresses not listed in the USPS Delivery Sequence File, and some newly constructed dwellings. While most AmeriSpeak households participate in surveys by web, non-internet households can participate in AmeriSpeak surveys by telephone. Households without conventional internet access but having web access via smartphones are allowed to participate in AmeriSpeak surveys by web. AmeriSpeak panelists participate in NORC studies or studies conducted by NORC on behalf of governmental agencies, academic researchers, and media and commercial organizations (https://amerispeak.norc.org/documents/Research/AmeriSpeak%20Technical%20Overview%202019%2002%2018.pdf**).**

Recruitment involves an initial stage in which sample units are invited to join AmeriSpeak online or by telephone in English or Spanish language. Study invitations are sent via postcard, a recruitment package, two follow-up post cards, and contact by NORC's telephone research center. In a second stage, a stratified random sub-sample of non-responders from the initial recruitment are targeted for non-response follow up. Consumer vendor data are matched to housing units and those that are flagged as having either a young adult (age 18-34 years) or minority (Hispanic and non-Hispanic African American) are oversampled for non-response follow up in addition to use of a stratified simple random sample based on consumer vendor data. A new recruitment package with an enhanced incentive offer is sent to these units

© 2020 Nong P et al. *JAMA Network Open*.

followed by face-to-face visits from NORC field interviewers to respondents' homes. Upon encouraging participation from these units, interviewers administer the recruitment survey in person or encourage respondents to register online or by telephone. Respondents are given a choice of either online or telephone participation in AmeriSpeak surveys. For example, in the 2014-2017 recruitment, 82% participants chose to receive online surveys while 18% selected telephone surveys.

NORC follows AAPOR reporting guidelines in calculation of response rate for panel-based research that takes into account all sources of non-response at each stage of the panel recruitment, management, and survey administration. NORC is also in compliance with the AAPOR Transparency Initiative and provided an in-depth profile of sample quality metrics for the study, the data collection field period, interview sample size, response rate statistics, design effect, and sampling margins of error in addition to other statistics.

© 2020 Nong P et al. *JAMA Network Open*.
